# Interactions between life expectancy and the incidence and mortality rates of cancer in China: a population-based cluster analysis

**DOI:** 10.1186/s40880-018-0308-x

**Published:** 2018-07-03

**Authors:** Xiuying Gu, Rongshou Zheng, Changfa Xia, Hongmei Zeng, Siwei Zhang, Xiaonong Zou, Zhixun Yang, He Li, Wanqing Chen

**Affiliations:** 1 Cancer Research Institute, Cancer Hospital, Xinjiang Medical University, Urumqi, 830011 P. R. China; 2 National Office for Cancer Prevention and Control, National Cancer Center/Cancer Hospital, Chinese Academy of Medical Sciences and Peking Union Medical College, Beijing, 100021 P. R. China

**Keywords:** Cancer, Incidence, Mortality, Life expectancy, China

## Abstract

**Background:**

The relationship between cancer and life expectancy is well established in both developed and developing countries. China is a vast country with significant geographical differences in population structure and healthcare, and thus provides a unique opportunity to analyze the complex relationship between life expectancy and cancer incidence and mortality rates.

**Methods:**

Cancer data were extracted for a total of 255 units (cities or counties) from the 2013 National Central Cancer Registry. Life expectancy data at the unit level were obtained from the National Centers for Disease Control and Prevention. Linear regression analysis was used to analyze the relationship between life expectancy and crude incidence and mortality rates of cancer. In a separate analysis, life expectancy was rated as low (< 76.0 years), middle (76–80 years), or high (> 80 years).

**Results:**

Overall, the cancer incidence and mortality rates positively correlated with life expectancy in both sexes (R at 0.37 and 0.50, *P *< 0.001). The correlation was significant for the following cancers: lung, colorectal, prostate, bladder and pancreas, as well as for lymphoma in men (R 0.36–0.58, *P *< 0.001), lung, breast, colorectal, thyroid, uterus, and ovary in women (R 0.18–0.51, *P *< 0.001). We failed to observe an association between upper gastrointestinal cancer and life expectancy. The number of cities/counties with low, middle and high life expectancy levels were 110, 101 and 44, respectively. The highest age-standardized cancer incidence rate was observed in areas with a high life expectancy level (192.83/100,000). The highest age-standardized mortality rate was in areas with the lowest life expectancy (118.44/100,000). Cancers of the stomach, liver and esophagus are major cancer types in areas with low and middle life expectancy. In contrast, areas with high life expectancy had high incidence and mortality rates of colorectal cancer, breast cancer in women and prostate cancer in men.

**Conclusions:**

Longer life expectancy is associated with higher overall cancer incidence and mortality in China. The cancer pattern also varies substantially across areas with different life expectancy levels. Life expectancy levels must be considered when developing strategies to prevent and treat cancers.

## Introduction

Cancer is a major public health problem worldwide [1, 2]. The majority of cancer types are more common in elderly populations. The relationship between cancer and life expectancy in the general population has been extensively studied [3–6]. The results provided consistent evidence for differing cancer profiles across life expectancy levels. In particular, cancer is associated with an aging population and socioeconomic development. Aging populations have led to major changes in the structure of the global population and in the scale of the cancer problem worldwide [7]. The United Nations Development Program (UNDP) has now incorporated life expectancy as a component of the Human Development Index (HDI) to evaluate the influence of social development on health issues (including cardiovascular diseases and cancer) across different countries [8–10].

Global life expectancy has increased by 5 years since 2000, with an increasingly narrower gap between high- and low-income countries [8]. In China, the average life expectancy in 2015 was 76.34 years (73.64 for men and 79.43 for women); up 5 years from 2000 [11]. Despite the rapidly increasing life expectancy, overall cancer incidence has been relatively stable, with decreasing age-standardized mortality rates in both men and women [2, 12–14]. The cancer pattern in China, however, is rather different from developed countries [15]. Also, China is a vast country with significant differences in population structure and healthcare, and thus provides a unique opportunity to analyze the complex relationship between cancer incidence, mortality and life expectancy, with potential relevance on a global scale.

## Materials and methods

### Data source

**Fig. 1:**
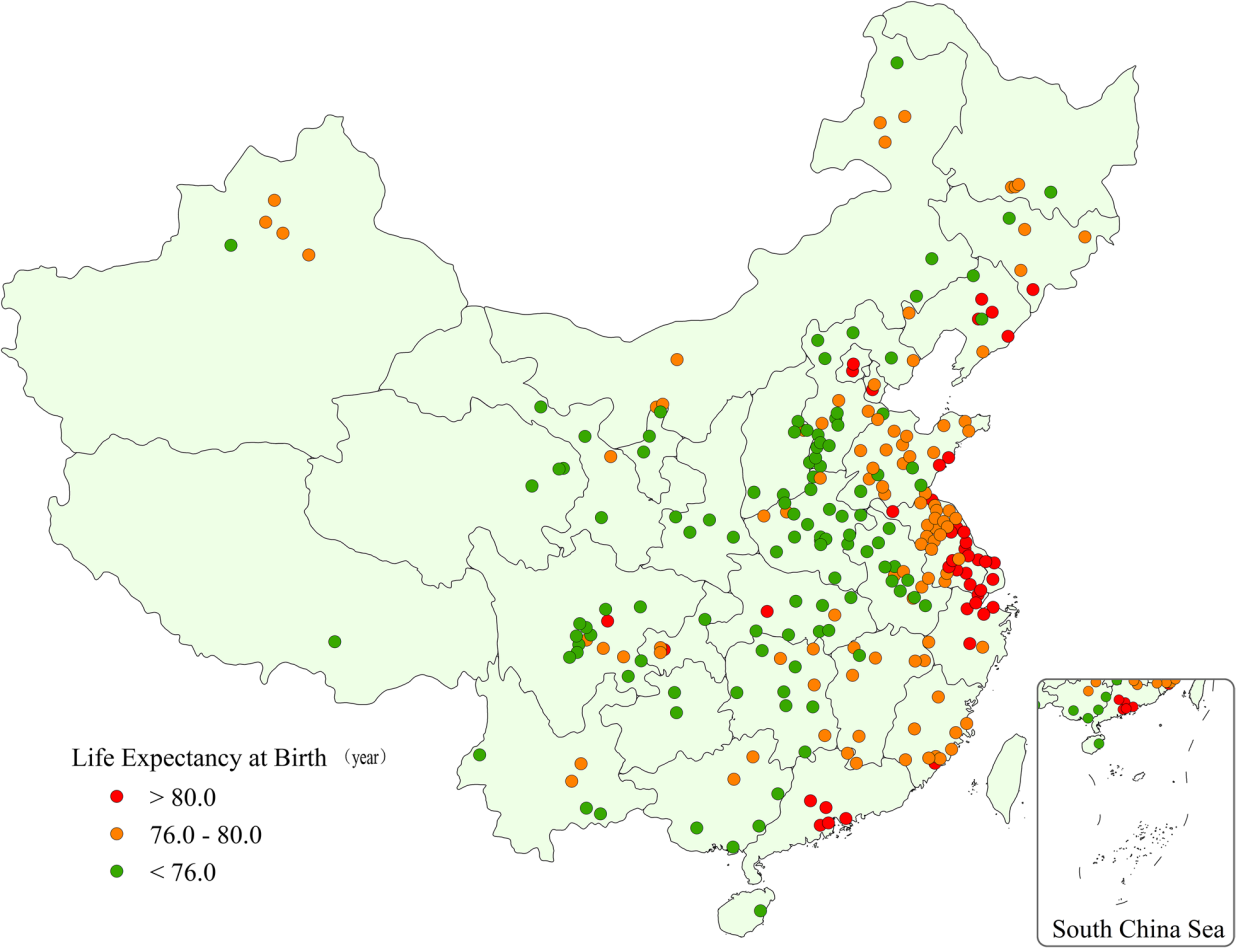
Map of the units (cities and counties) included in data analysis. Red: high life expectancy level units (life expectancy > 80 years); orange: middle life expectancy level units (life expectancy 76.0–80.0 years); green: low life expectancy level units (life expectancy < 76.0 years)

Cancer data were retrieved from the National Central Cancer Registry (NCCR). A total of 255 units (cities or counties) were included in the analysis, that covered 31 provinces, 88 cities and 167 counties. The total population covered by NCCR was 226,494,490 (114,860,339 men and 111,634,151 women) and accounted for 16.65% of the national population at the end of 2013. Life expectancy data were obtained from the National Centers for Disease Control and Prevention (NCDC). Life expectancy was low (< 76 years) in 110 units, 101 units in the middle (76–80 years) and high (> 80 years) in 44 units. Life expectancy levels, based on geographical location, are shown in Fig. 1.

### Quality control

Cancer data were collected, audited and maintained by the NCCR according to the standards set forward by the “*Guideline for Chinese Cancer Registration*”, “Cancer Incidence in Five Continents Volume IX”, as well as relevant data quality criteria by the International Agency for Research on Cancer/International Association of Cancer Registries (IARC/IACR) [16]. The assessments of quality measures include, but are not limited to, the proportion of morphologic verification (MV%), the percentage of cancer cases identified with death certification only (DCO%), the mortality (M) to incidence (I) ratio (M/I), the percentage of uncertified cancers (UB%). The MV%, DCO%, and M/I ratio of overall indicators in this analysis were 68.04%, 1.74% and 0.62%, respectively.

#### Statistical analysis

For descriptive analysis, the 255 units (cities/counties) were divided into three categories and based on the average life expectancy based on the 2009 report by the National Population Census [11]: low, middle, and high using 76 and 80 years of age as the cutoff points. A linear regression model (considering life expectancy as a continuous variable) was used to estimate the relationship between crude incidence and mortality rates of cancer and life expectancy using the data for each city/county. A sex-stratified analysis was conducted. A separate set of correlation analyses were conducted to analyze the relationship between each of the top 10 cancers with life expectancy. The Chinese population in 2000 and the World Segi’s population were used to calculate age-standardized rates. Softwares for data checking and evaluation included MS-Excel, IARCcrgTools2.05 issued by International Agency for Research on Cancer (IARC) and International Association of Cancer Registries (IACR) [17]. All statistical analyses were conducted using SAS (SAS Institute Inc.; Cary, NC, USA).

## Results

### Linear regression analysis between cancer data and life expectancy

**Fig. 2:**
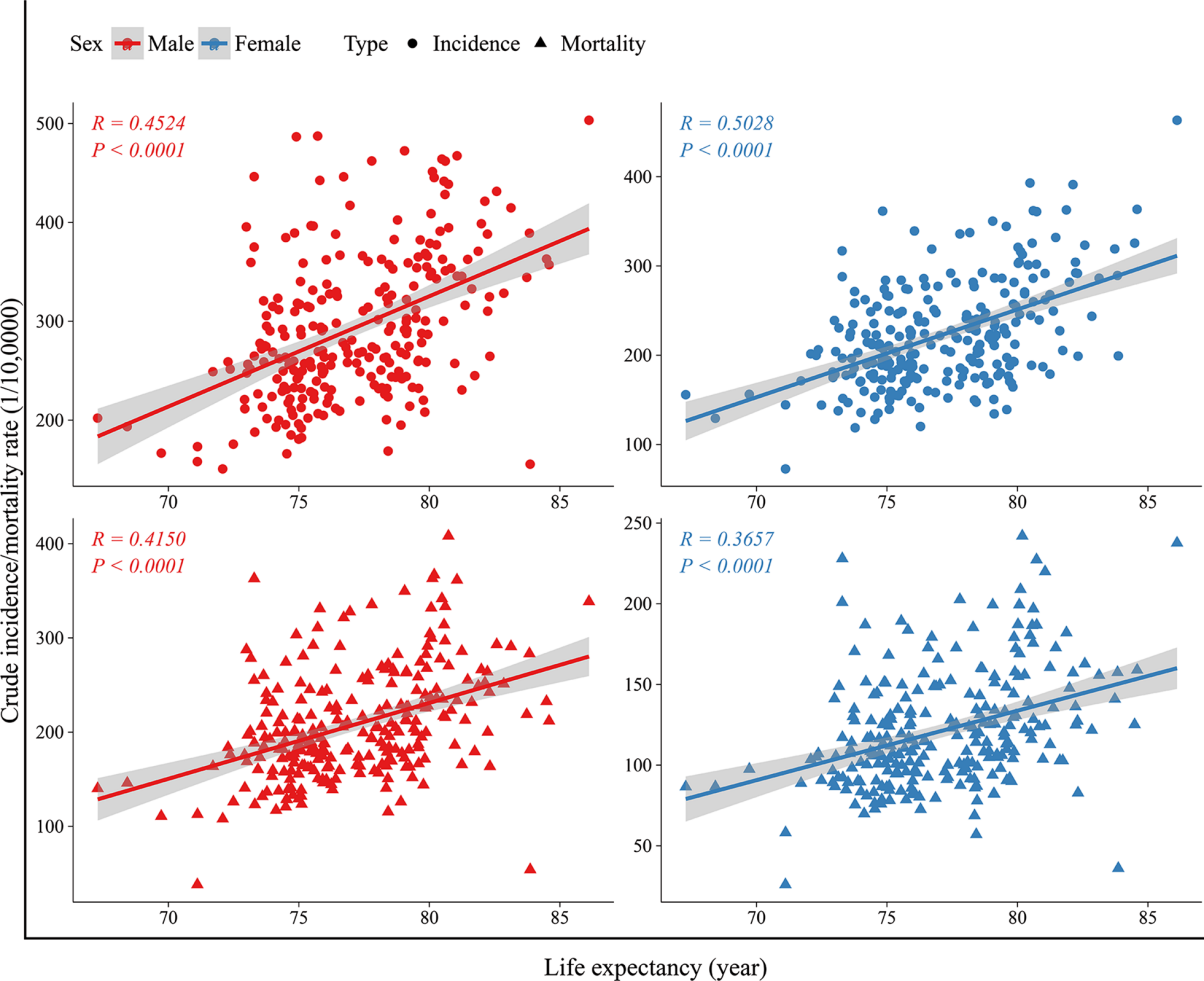
Correlation between life expectancy and incidence/mortality of overall cancer, stratified by sex

In both men and women, life expectancy correlated positively with both overall cancer incidence (*R*_*male incidence*_ =* 0.45, R*_*female incidence*_ =* 0.50*, *P *<* 0.001* for both men and women) and mortality (*R*_*male mortality*_ =* 0.42, R*_*female mortality*_ =* 0.37*, *P *<* 0.001* for both men and women) (Fig. 2).

**Fig. 3:**
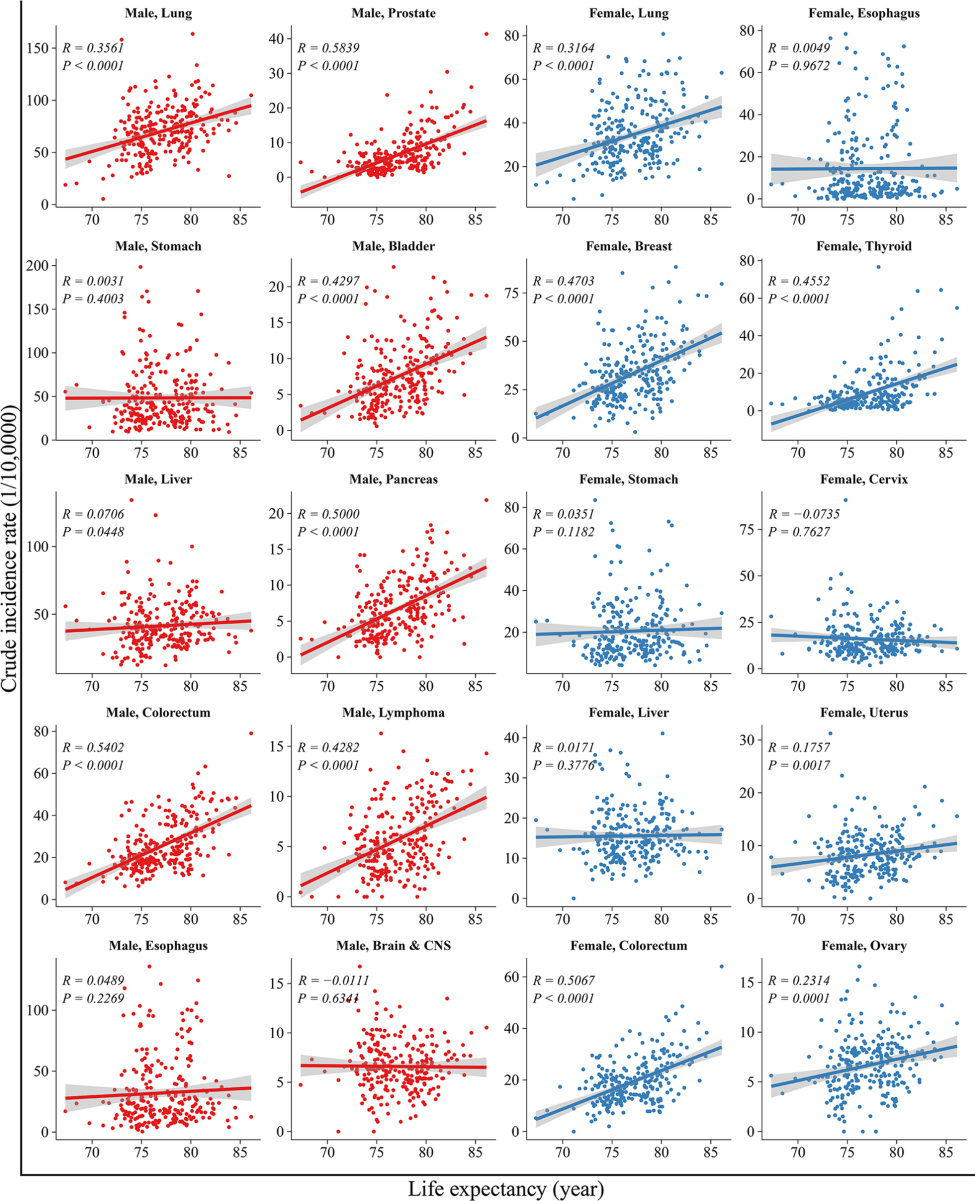
Correlation between life expectancy and incidence rates for the top ten leading types of cancers, stratified by sex. The top ten leading types of cancer incidence were published by overall cancer registration data in 2013 and published previously on *Cancer Letters* (Chen W, Zheng R, Zhang S, et al. Cancer incidence and mortality in china, 2013 [12]. Cancer Lett. 2017;401:63–71)

The correlation between life expectancy and the incidence of the top 10 cancers are shown in Fig. 3. In men, life expectancy correlated positively with the incidence of cancers in the lungs (*R *=* 0.36, P *<* 0.001*), colorectum (*R *=* 0.54, P *<* 0.001*), prostate (*R *=* 0.58, P *<* 0.001*), bladder (*R *=* 0.43, P *<* 0.001*), pancreas (*R *=* 0.50, P *<* 0.001*) and lymphoma (*R *=* 0.43, P *<* 0.001*). The correlation was most robust for prostate cancer (*R *=* 0.58*, *P *<* 0.001*). Life expectancy did not correlate with cancers of the stomach, liver, esophagus, brain and central nervous system (CNS). In women, life expectancy correlated positively with the incidence of cancers in the lungs, breast, colorectum, thyroid, uterus and ovary (*R* from 0.18 to 0.51, all *P *<* 0.01*). The correlation was most robust for colorectal cancer (*R *=* 0.51*, *P *<* 0.001*).

**Fig. 4:**
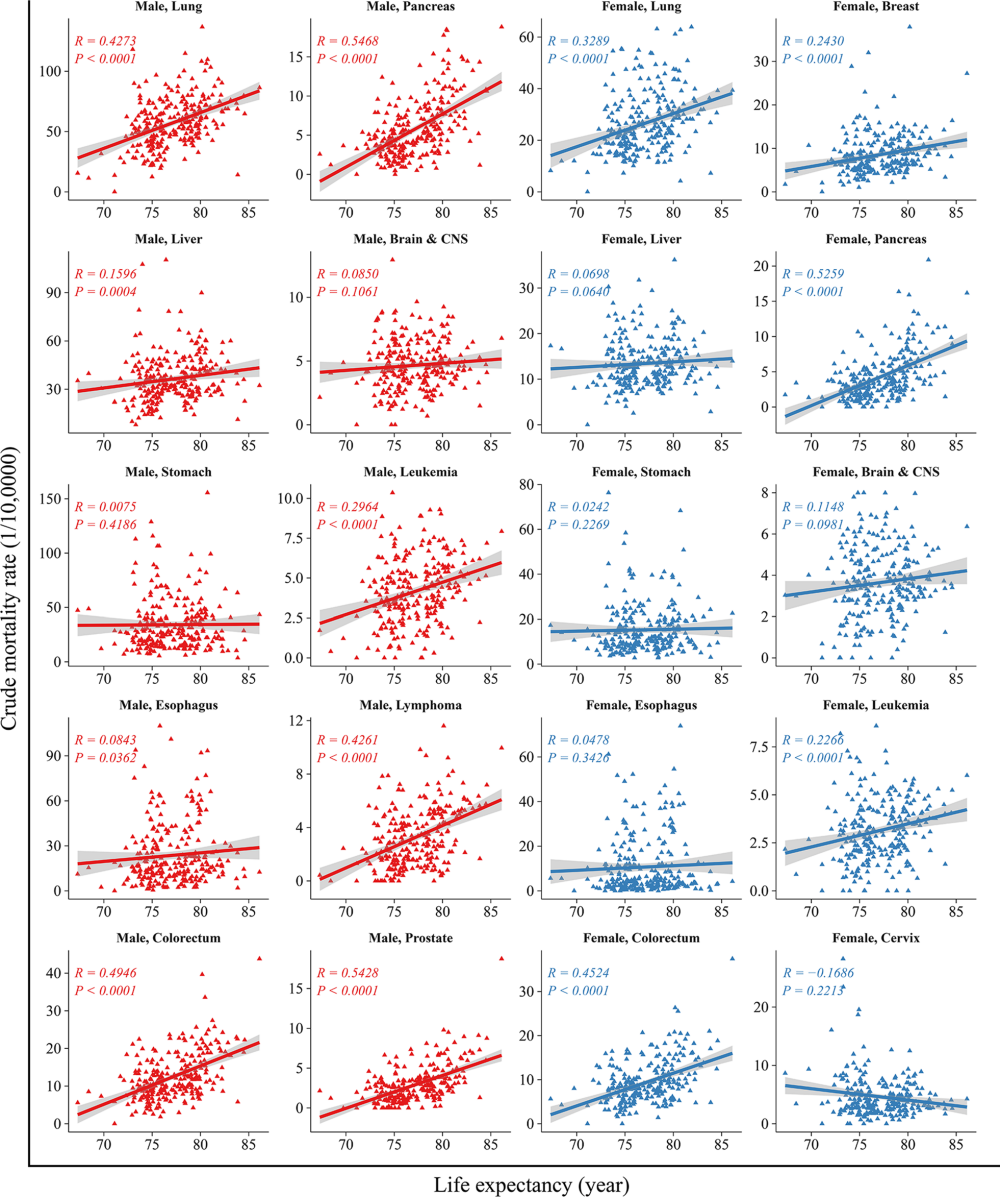
Correlation between life expectancy and mortality rates for the top ten leading types of cancers, stratified by sex. The top ten leading types of cancer death were published by overall cancer registration data in 2013 and published previously on *Cancer Letters* (Chen W, Zheng R, Zhang S, et al. Cancer incidence and mortality in china, 2013 [J]. Cancer Lett. 2017;401:63–71)

**Fig. 5:**
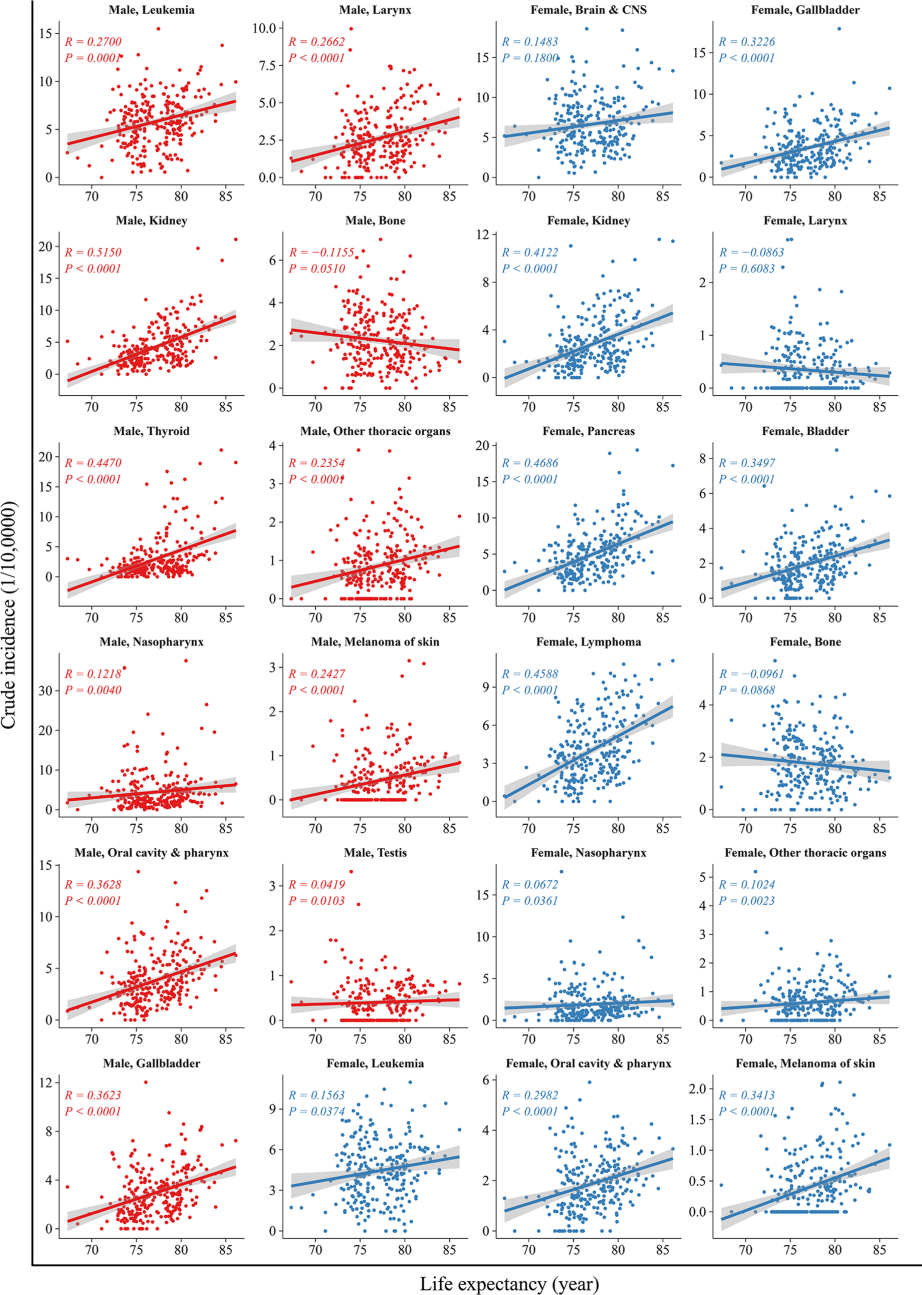
Correlation between life expectancy and incidence rates in others cancers, stratified by sex

**Fig. 6:**
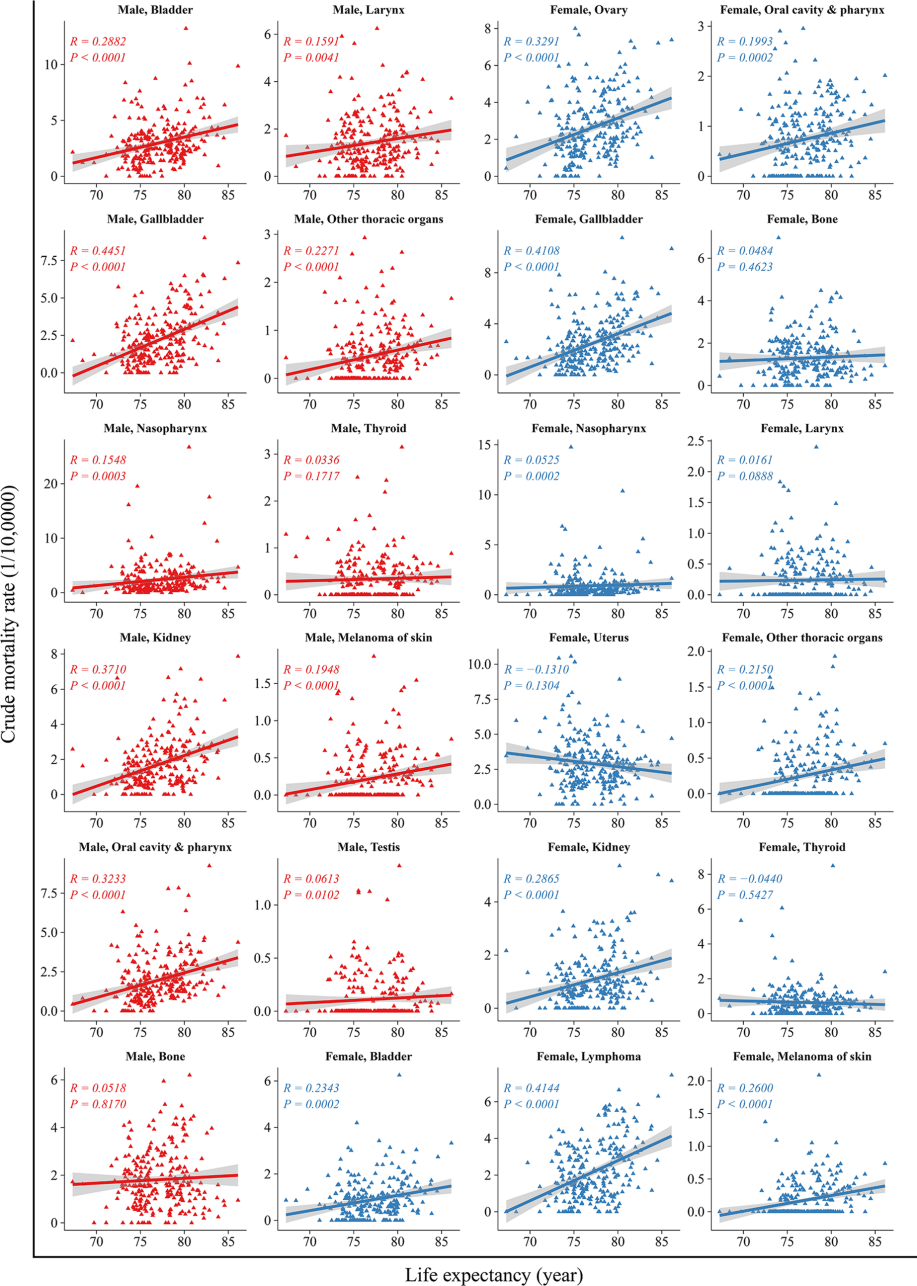
Correlation between life expectancy and mortality rates in others cancers, stratified by sex

The correlation between life expectancy and cancer mortality of the top 10 cancers are shown in Fig. 4. In men, life expectancy was correlated positively with the mortality in cancers of the lungs, liver, colorectum, pancreas, prostate, leukemia, and lymphoma (*R* from 0.16 to 0.55, all *P *<* 0.05*), but not cancers of the upper gastrointestinal tract (*R *=* 0.01, P *=* 0.419*). The correlation was most robust for pancreatic cancer (*R *=* 0.55*, *P *<* 0.001*). In women, life expectancy correlated positively with the mortality in cancers of the lungs, colorectum, breast, pancreas and leukemia (*R* from 0.23 to 0.53, all *P *<* 0.05*), but not cancers of the liver, stomach, esophagus, brain and CNS and cervix. The association between life expectancy and less common types of cancer varied substantially. Such as, in both sexes, life expectancy correlated positively with the incidence of oral, nasopharynx, gallbladder, kidney and melanoma of the skin cancers (*R* from 0.07 to 0.52, all *P* < 0.05). And there were the same associations between life expectancy and mortality in those cancers (*R* from 0.05 to 0.45, all *P* < 0.05) (Figs. 5, 6).

### Incidence

#### Overall incidence

Overall cancer incidence rates in the areas with different life expectancy levels are shown in Table 1. Areas with high life expectancy (> 80 years) had a high crude incidence rate, followed by areas with middle (76–80 years) and low (< 76 years) life expectancy levels. There was a U-shaped association between age-standardized incidence rates and life expectancy levels: areas with a middle life expectancy level had the lowest age-standardized incidence rate by world standard population (ASIRW). The patterns of crude incidence rates in men and women were similar to the overall sample that included both sexes. However, after adjusting for age, cancer incidence rates in men in low life expectancy level areas was higher than that in high and middle level areas. In women, the adjusted incidence rate was much higher in high life expectancy level areas than that of low and middle life expectancy level areas.

**Table 1:** Top 10 cancer incidence in three life expectancy levels areas of China, 2013

Rank	Gender	Low life expectancy level areas				Middle life expectancy level areas					High life expectancy level areas			
		Site	Incidence	Proportion	ASR^a^	Site	Incidence	Proportion	ASR^a^		Site	Incidence	Proportion	ASR^a^
			(1/10^5^)	(%)	(1/10^5^)		(1/10^5^)	(%)	(1/10^5^)			(1/10^5^)	(%)	(1/10^5^)
0	Both	All sites	242.77	100.00	186.91	All sites	262.81	100.00	179.00		All sites	339.61	100.00	192.83
1		Lung	49.39	20.34	37.41	Lung	55.64	21.17	36.88		Lung	66.44	19.56	34.90
2		Stomach	37.50	15.45	28.76	Stomach	32.36	12.31	21.66		Colorectum	38.42	11.31	20.57
3		Liver	29.17	12.01	22.25	Liver	27.42	10.43	18.56		Stomach	31.83	9.37	17.08
4		Esophagus	26.87	11.07	20.58	Esophagus	24.42	9.29	16.27		Breast	56.75	8.35	34.59
5		Colorectum	17.85	7.35	13.60	Colorectum	22.61	8.60	15.12		Liver	28.08	8.27	15.83
6		Breast	28.36	5.67	21.26	Breast	37.05	6.94	25.18		Thyroid	17.54	5.16	12.19
7		Cervix	16.24	3.25	12.19	Thyroid	8.17	3.11	5.89		Esophagus	16.14	4.75	8.55
8		Brain, CNS	6.79	2.80	5.53	Cervix	14.21	2.66	9.71		Pancreas	10.16	2.99	5.21
9		Leukemia	4.83	1.99	4.46	Brain, CNS	6.21	2.36	4.63		Brain, CNS	8.81	2.59	5.82
10		Pancreas	4.03	1.66	3.06	Pancreas	6.10	2.32	4.04		Lymphoma	8.74	2.57	5.21
0	Men	All sites	276.05	100.00	220.73	All sites	294.24	100.00	207.57		All sites	365.39	100.00	209.60
1		Lung	65.90	23.87	52.32	Lung	73.76	25.07	51.26		Lung	86.09	23.56	47.22
2		Stomach	51.72	18.74	41.26	Stomach	44.90	15.26	31.41		Colorectum	43.61	11.94	24.27
3		Liver	41.18	14.92	32.42	Liver	39.91	13.56	27.97		Stomach	43.53	11.91	24.21
4		Esophagus	35.64	12.91	28.47	Esophagus	33.74	11.47	23.61		Liver	41.14	11.26	24.16
5		Colorectum	19.84	7.19	15.75	Colorectum	25.67	8.72	17.87		Esophagus	23.95	6.55	13.38
6		Brain, CNS	6.97	2.52	5.79	Bladder	7.81	2.66	5.36		Prostate	16.80	4.60	8.51
7		Bladder	5.37	1.94	4.21	Pancreas	6.95	2.36	4.83		Bladder	13.24	3.62	7.08
8		Leukemia	5.29	1.92	4.97	Prostate	6.72	2.28	4.48		Pancreas	11.26	3.08	6.13
9		Lymphoma	4.72	1.71	3.89	Lymphoma	6.17	2.10	4.53		Kidney	10.23	2.80	6.04
10		Pancreas	4.56	1.65	3.61	Leukemia	6.00	2.04	5.24		Lymphoma	9.96	2.72	6.12
0	Women	All sites	207.45	100.00	154.66	All sites	230.36	100.00	152.91		All sites	313.81	100.00	178.58
1		Lung	31.86	15.36	22.94	Breast	37.05	16.08	25.18		Breast	56.75	18.08	34.59
2		Breast	28.36	13.67	21.26	Lung	36.93	16.03	23.28		Lung	46.78	14.91	23.36
3		Stomach	22.41	10.80	16.31	Colorectum	19.46	8.45	12.51		Colorectum	33.24	10.59	17.10
4		Esophagus	17.58	8.47	12.79	Stomach	19.42	8.43	12.32		Thyroid	26.34	8.39	18.22
5		Liver	16.42	7.91	11.98	Esophagus	14.81	6.43	9.18		Stomach	20.13	6.41	10.37
6		Cervix	16.24	7.83	12.19	Liver	14.54	6.31	9.27		Cervix	15.25	4.86	9.74
7		Colorectum	15.73	7.58	11.50	Cervix	14.21	6.17	9.71		Liver	15.02	4.79	7.67
8		Uterus	7.75	3.74	5.84	Thyroid	12.74	5.53	9.12		Uterus	12.02	3.83	7.14
9		Brain, CNS	6.60	3.18	5.28	Uterus	8.31	3.61	5.66		Brain, CNS	9.71	3.09	6.18
10		Ovary	6.12	2.95	4.78	Ovary	7.26	3.15	5.11		Ovary	9.17	2.92	5.77

^a^ASR: age-standardized incidence rate (Segi’s Standard Population)

#### Top ten leading cancer types

Incidence rates for the top 10 cancers in the areas with different life expectancy levels are shown in Table 1. The overall analysis showed that lung cancer is the most common cancer in both sexes. ASIRW was significantly higher in low life expectancy areas (37.41/100,000) than in high life expectancy level areas (34.90/100,000). Cancers of the stomach, liver, and esophagus were all in the top 5 list in both low and middle life expectancy level areas. Similar to that of lung cancer, the ASIRW in middle life expectancy areas were lower than in low life expectancy areas (21.66 vs. 28.76/10,000, 18.56 vs. 22.25/10,000, and 16.27 vs. 20.58/10,000) for the three types of cancers, respectively. In high life expectancy level areas, colorectal cancer ranked second which was higher than middle and low life expectancy areas. And incidence rates of breast and thyroid cancers in women increased stepwise from low to high expectancy level areas.

Cancer types varied significantly among areas with different life expectancy. In low and middle life expectancy level areas, the most common cancers in men were the cancers of the lung, stomach, liver and esophagus. In high life expectancy level areas, colorectal cancer was also very common; the ASIRW of prostate and bladder cancers was also higher than in low and middle level areas. In women, cancers of the lung, breast, stomach, esophagus and liver were the most common types in low life expectancy level areas. In middle life expectancy level areas, cancers of the breast, lung, colorectum, stomach, and esophagus were the most common cancers in women. In high life expectancy level areas, cancers of the breast, lung, colorectum, thyroid and stomach were the most common cancers.

### Mortality

#### Overall mortality

Cancer mortality rates in areas with different life expectancy levels are shown in Table 2. High life expectancy level areas had higher crude mortality rates, followed by middle and then low life expectancy level areas. Stratified analysis based on sex produced similar findings: the crude cancer mortality rate in high life expectancy level areas was higher than that in middle and low-level areas. However, the age-standardized mortality rate correlated negatively with life expectancy, with the low life expectancy level areas having the highest age-standardized mortality rate by world standard population (ASMRW). The patterns of ASMRW in men and women were similar to the overall analysis that included both men and women.

**Table 2:** Top 10 cancer mortality in three life expectancy levels areas of China, 2013

Rank	Gender	Low life expectancy level areas				Middle life expectancy level areas				High life expectancy level areas			
		Site	Mortality	Proportion	ASR^a^	Site	Mortality	Proportion	ASR^a^	Site	Mortality	Proportion	ASR^a^
			(1/10^5^)	(%)	(1/10^5^)		(1/10^5^)	(%)	(1/10^5^)		(1/10^5^)	(%)	(1/10^5^)
0	Both	All sites	156.51	100.00	118.44	All sites	167.98	100.00	110.99	All sites	200.41	100.00	103.08
1		Lung	38.07	24.32	28.45	Lung	46.05	27.41	29.90	Lung	54.93	27.41	27.43
2		Stomach	26.56	16.97	19.93	Liver	24.56	14.62	16.44	Liver	24.85	12.4	13.61
3		Liver	24.70	15.78	18.73	Stomach	23.32	13.88	15.15	Stomach	22.91	11.43	11.42
4		Esophagus	19.21	12.28	14.38	Esophagus	18.21	10.84	11.77	Colorectum	18.53	9.25	8.94
5		Colorectum	8.75	5.59	6.46	Colorectum	10.94	6.51	7.04	Esophagus	13.02	6.50	6.53
6		Brain, CNS	4.14	2.64	3.35	Pancreas	5.36	3.19	3.51	Pancreas	9.69	4.83	4.86
7		Breast	8.48	2.63	6.30	Breast	9.12	2.67	5.99	Breast	12.26	3.06	6.54
8		Leukemia	3.22	2.06	2.92	Brain, CNS	4.19	2.49	3.09	Leukemia	4.87	2.43	3.20
9		Pancreas	3.18	2.03	2.38	Leukemia	3.86	2.30	3.09	Lymphoma	4.85	2.42	2.61
10		Cervix	4.93	1.53	3.69	Lymphoma	3.00	1.79	2.06	Gallbladder	4.67	2.33	2.24
0	Men	All sites	194.79	100.00	154.68	All sites	210.95	100.00	146.69	All sites	247.26	100.00	134.05
1		Lung	51.47	26.43	40.63	Lung	61.67	29.23	42.34	Lung	73.50	29.73	38.95
2		Stomach	36.02	18.49	28.50	Liver	35.78	16.96	24.93	Liver	36.47	14.75	20.98
3		Liver	35.08	18.01	27.63	Stomach	32.11	15.22	22.16	Stomach	30.98	12.53	16.33
4		Esophagus	25.50	13.09	20.26	Esophagus	24.98	11.84	17.23	Colorectum	20.80	8.41	10.76
5		Colorectum	9.85	5.05	7.73	Colorectum	12.40	5.88	8.51	Esophagus	19.05	7.70	10.26
6		Brain, CNS	4.60	2.36	3.84	Pancreas	6.01	2.85	4.16	Pancreas	10.72	4.33	5.77
7		Pancreas	3.67	1.89	2.91	Brain, CNS	4.53	2.15	3.45	Prostate	6.69	2.71	3.09
8		Leukemia	3.63	1.86	3.31	Leukemia	4.34	2.06	3.55	Lymphoma	5.69	2.30	3.22
9		Lymphoma	2.53	1.30	2.06	Lymphoma	3.62	1.72	2.58	Leukemia	5.62	2.27	3.74
10		Bladder	2.50	1.28	1.95	Bladder	3.10	1.47	2.09	Brain, CNS	4.92	1.99	3.28
0	Women	All sites	115.89	100.00	83.64	All sites	123.62	100.00	77.46	All sites	153.53	100.00	74.37
1		Lung	23.84	20.57	16.78	Lung	29.92	24.21	18.28	Lung	36.35	23.68	16.76
2		Stomach	16.53	14.26	11.6	Stomach	14.24	11.52	8.57	Colorectum	16.26	10.59	7.28
3		Liver	13.69	11.81	9.81	Liver	12.97	10.49	8.10	Stomach	14.83	9.66	6.91
4		Esophagus	12.54	10.82	8.71	Esophagus	11.23	9.09	6.57	Liver	13.22	8.61	6.43
5		Breast	8.48	7.31	6.30	Colorectum	9.43	7.63	5.68	Breast	12.26	7.99	6.54
6		Colorectum	7.58	6.54	5.29	Breast	9.12	7.38	5.99	Pancreas	8.65	5.64	3.98
7		Cervix	4.93	4.25	3.69	Pancreas	4.69	3.80	2.89	Esophagus	6.99	4.55	2.99
8		Brain, CNS	3.64	3.14	2.87	Brain, CNS	3.84	3.11	2.71	Gallbladder	4.97	3.24	2.20
9		Uterus	3.22	2.78	2.38	Cervix	3.65	2.96	2.38	Ovary	4.62	3.01	2.51
10		Leukemia	2.80	2.41	2.53	Leukemia	3.36	2.71	2.64	Leukemia	4.13	2.69	2.70

^a^ASR: age-standardized mortality rate (Segi’s Standard Population)

#### Top ten leading cancer types

Mortality rates for the top 10 leading cancers in areas with different life expectancy levels are shown in Table 2. Lung cancer was the leading cause of cancer death regardless of life expectancy levels in both men and women. Other cancer types with high mortality rates included cancers of the stomach, liver, esophagus and colorectum. The ASMRW of colorectal and pancreatic cancers in high life expectancy level areas was significantly higher than that in low and middle level areas.

The pattern of ASMRW in men was similar to that in the overall analysis that included both sexes. In low and middle life expectancy level areas, stomach cancer ranked second in cancer mortality in women, followed by liver and esophageal cancers. The mortality of cervical cancer in low life expectancy level areas was higher than that in other areas. The ASMRW of colorectal and breast cancers in women was higher in high life expectancy level areas than that in low and middle level areas.

## Discussion

Life expectancy was adopted by the United Nations General Assembly as an important domain of the Sustainable Development Goals (SDGs) in 2015 [8]. It provides an estimate of the average expected life span under certain conditions, based on current mortality rates. It is the most representative and comprehensive index to judge the social economy and healthcare development of a country or region. Differences in life expectancy were significant across different levels of socioeconomic status, including income [18], education [19] and health services [20].

In the present study, 255 geographical units (cities or counties) were divided into 3 levels based on life expectancy: high, middle and low. The analysis showed the highest crude cancer incidence and mortality rate in high life expectancy level areas, whereas the low life expectancy level areas had the lowest crude cancer incidence and mortality. Areas with higher life expectancy typically have higher incidence and relatively lower mortality [21]. In the present study, the high crude cancer incidence in high life expectancy level areas could be mainly attributed to the high incidence of breast and colorectal cancers (Table 1). The incidence of these cancers is relatively low in less developed and developing countries, but is expected to rise with increasing life expectancy, urbanization, and the adoption of a western lifestyle [22, 23].

Consistent with previous studies [24–26], longer life expectancy was associated with a variety of cancers in the present study, including colorectal, breast, thyroid, prostate and bladder cancers. Coinciding with the transforming cancer trends, these cancers constitute a large burden of disease with the aging population in China [12]. China has undergone rapid demographic and epidemiological changes in the past few decades, including striking declines in fertility and increases in life expectancy at birth [27]. The increase in life expectancy is a key driver of years of life lost (YLLs) and the increases of future burden of cancer [28]. The WHO reported that high income countries (e.g., Japan, Switzerland, Singapore and the US) had an average life expectancy of 80 years or more, but low-middle income countries have greater annual increases in life expectancy [8]. The residents of countries with high living standards have lower mortality rates [1]. In the US, overall cancer death rates have decreased by 25% over the past 2 decades [29]. In China, overall cancer mortality continues to decline while cancer incidence remains relatively stable [2, 28]. In this study, we found highest the ASIRW and the lowest ASMRW in high life expectancy level areas. In contrast, low life expectancy level areas had the highest ASMRW.

Lung cancer remains the most commonly diagnosed cancer and the leading cause of cancer deaths. The incidence of lung cancer was positively associated with life expectancy. Underlying risk factors remain unknown, but could include factors other than ageing itself, including cigarette smoking, air pollution and radon exposure, as previously suggested by epidemiological studies [30, 31]. With the tobacco epidemic shifting to less developed areas, lung cancer incidence is also increasing in developing regions. Cigarette smoking in China has increased substantially since the 1980s [32]. Smoking is the most important risk factor associated with rising death risks, with 50% of the five million smoking-related deaths worldwide occurring in low- and middle-income countries, and 80% of which are men [33]. In China, the higher rate of smoking was not only in men, but also especially in rural residents [34]. Household air pollution may be another main reason for lung cancer in China, especially in rural areas with the use solid fuels [35].

Breast cancer is the most commonly diagnosed cancer in Chinese women and the disease burden has experienced a rapid growth over the last decade [2, 36]. There is a direct, strong, and meaningful correlation between life expectancy and standardized breast cancer incidence and mortality rates [4]. The age-standardized incidence rate of breast cancer in high life expectancy level areas was 1.6 times as much as that in low life expectancy level areas. Such difference could possibly be attributed to westernization and differences in age at menarche, number of completed births, and other reproductive and hormonal factors [3, 37].

In a previous study, the incidence of colorectal cancer was 6–7 times higher in regions with very high versus low HDI in both sexes [6]. It has been considered one of the clearest markers of transition, as increases in colorectal cancer incidence have generally paralleled increases in human development across most countries [12, 38]. In China, the ASIR of colorectal cancer in urban areas has been reported to be 52% higher than in rural areas [39]. We also observed a positive correlation between life expectancy and the incidence rates of prostate, and pancreatic cancers, but the significance of such association is obscure due to the relatively low incidence rates of these cancers in China [29, 40, 41]. Besides, the incidence of thyroid cancer in high life expectancy level areas was also higher than that in low and middle level areas, likely reflecting improved diagnosis and treatment. Over-diagnosis of thyroid cancer, however, could have also contributed to the association [42, 43]. A finding distinct from that reported in western countries is the fact that cancers of the stomach and liver were major causes of death regardless of life expectancy levels. In contrast to a positive correlation between life expectancy and stomach cancer incidence rate reported by others [25], we did not find any significant correlation between life expectancy and either the incidence or mortality rates of the cancers of the stomach or liver.

The highest ASIR occurred in low life expectancy regions in men, but in high life expectancy regions in women. Such difference could be partially attributed to the higher incidence rates of most common cancers (such as lung, stomach, liver and esophageal cancers) in men in low life expectancy areas (versus in the high life expectancy areas). Factors that influence life expectancy, including health-related behaviors (smoking, obesity, and exercise) and local area characteristics (education, income and government expenditure levels), produce robust impact on the development of cancers [18]. In women, the incidence of breast cancer significantly increased from low life expectancy level areas to high level areas. Ghoncheh’s study showed that the incidence of breast cancer increases with increasing life expectancy, increasing urbanization, and the adoption of a western lifestyle [4]. However, earlier detection may have contributed to the observed increase of incidence for both breast and thyroid cancers [33]. Evidently, the major burden of over diagnosis or overtreatment occurs in women [44]. It is noteworthy that cervical cancer incidence was much higher in low life expectancy level areas in the present study, most likely due to disparities in socioeconomic status and access to high-quality health care [45]. In the 2013 report by the NCCR, the highest mortality rate of cervical cancer was in the northwest and southern rural areas (4.4 per 100,000), with the lowest mortality in eastern urban and northeast rural areas (2.1 per 100,000) [12]. Such geographical distribution pattern is consistent with life expectancy differences across the country [46].

The strength of the present study is its wide coverage of geographical locations and socioeconomic status. The study included data from a total of 255 cities and counties across 31 provinces of China and represented a population of 226.5 million people. However, the sampling was not random. Also, the areas covered by the registry probably had disproportionately high levels of economic development levels, and thus longer life expectancy levels than the national average. Nevertheless, the cancer data used in this study represents the best available nationwide data in China. Moreover, we used the same methods to compare cancer incidence and mortality in three urbanization [47] and GDP [48] levels. These findings may provide an important basis for the next phase of HDI research.

## Conclusions

Longer life expectancy is associated with overall rising cancer incidence and mortality in China. However, there is a complex relationship between cancer patterns (incidence, mortality and types) and life expectancy. Ongoing trends, as reflected by differences among cities/counties with varying life expectancy, include a reduction in infection-related cancers (for example, stomach, liver and cervical cancers) and an increase in cancers linked to a western lifestyle (for example, breast and colorectal cancers). Strategic planning at the governmental level, including the appropriation of resources and programs must be a priority when considering these changes.

## Abbreviations

NCCR: National Central Cancer Registry
ASIR: age-standardized incidence rate
ASMR: age-standardized mortality rate
HDI: Human Development Index
UNDP: United Nations Development Program
NCDC: National Centers for Disease Control and Prevention
IARC/IACR: International Agency for Research on Cancer/International Association of Cancer Registries
CI5: Cancer Incidence in Five Continents
